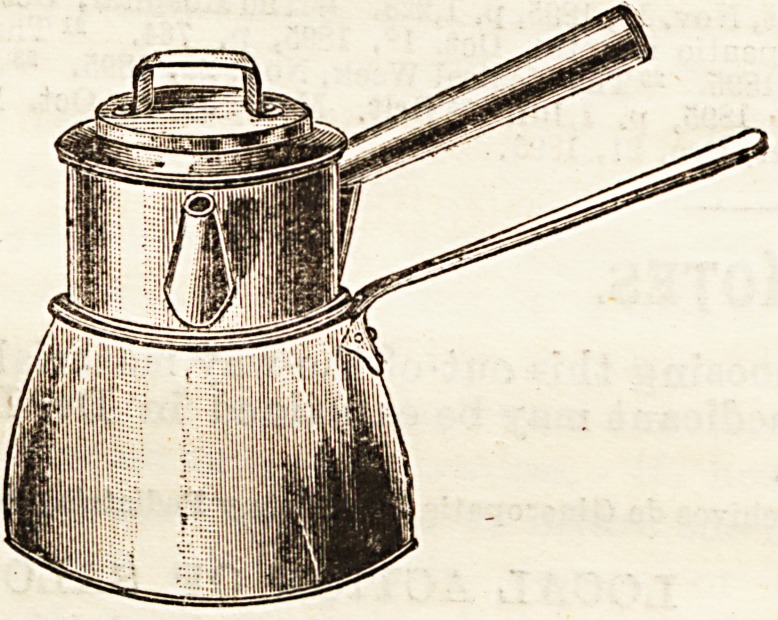# New Appliances and Things Medical

**Published:** 1896-04-18

**Authors:** 


### NEW APPLIANCES AND THINGS MEDICAL.

### THE IZAL AUTOMATIC DISINFECTOR.

(Newton, Chambers, and Co., Thorncliffe, Sheffield.)

This is a new and ingenious device for ensuring the proper disinfection of closets, sinks, traps, or ventilation chambers. It is, however, more particularly intended for the ordinary closets in private houses which are provided with a water supply. The method is as follows: Into the service pipe is screwed one end of a syphon tube, the other end of which dips into a convenient receptacle for the disinfectant. By means of a tap and valve the amount of the fluid disinfectant which passes into the service pipe, and consequently into the pan of the closet, each time the plug is pulled or water flows from the pipe can be regulated according to requirements. The whole apparatus, which is simplicity itself, costs only 5s. The chief merits are its automatic working and its visibility, and the fact that it can be applied by anyone without the assistance of a plumber. The receptacle for the disinfectant is made of transparent glass, so that, being open to inspection, as soon as the fluid becomes nearly exhausted the supply can be replenished. The apparatus was made for the automatic supply of Izal to closets, and probably no other disinfectant will answer the purpose more completely than this powerful germicide. For hospitals, institutions, and public lavatories such a contrivance has many obvious advantages.

### AYMARD'S PATENT MILK STERILISER.

(Aymard's Patent Steriliser Company, 19a, Coleman Street, S.E.)

There is no doubt that the only practicable way of rendering milk sterile is by heat. As milk flows from the healthy cow's udder it is germ-free, but it is quite out of the question to believe that it can be collected, transported, and then distributed by the retail dealer, without running great risk of contamination with microbes, which, even though they may in many cases not be definite disease-germs, are still capable of spoiling the milk as a food, especially for infants. It is well recognised that so far as living bacilli are concerned bringing the milk to the boil even for an instant is sufficient to destroy their vitality. But the art of boiling milk is not quite so simple as some may think. It is easy enough to make it boil over, and it is easy enough to burn it by making one side of the pan too hot, but neither of these gives any assurance that the whole mass of the milk has been raised to the proper temperature. For this purpose it is necessary that the heat should be applied all over the vessel containing the milk. The Aymard's milk steriliser consists of a vessel within a vessel; the inner one, which has a separate lid, containing the milk, the outer one containing water below and steam above, the advantages over the ordinary milk boilers being that the heat is equally applied to every part of the inner vessel, and that this being of metal, the heat is quickly transmitted to the milk, which can thus be sterilised without that prolonged heating which is largely the cause of the peculiar taste of boiled milk, and also without the cream separating. The construction offers various advantages. Thus, the milk vessel can be removed, and the milk poured out without taking off either the outer or the inner lid; this is found in practice to be a very great benefit. The apparatus is made in various sizes, ranging from three-quarters of a pint to six gallons, and is certainly an effectual arrangement for the purposes in view. We think, however, that the moderately small sizes are the best, as the time occupied in warming large quantities must be considerable, and must detract from one of the advantages claimed.

### BERMALINE BREAD, INFANT RUSKS, MALT EXTRACT, &c.

(Montgomerie and Co., Limited, Partick, Glasgow.)

We have received samples of the above preparations. With regard to the first-mentioned, namely, the bermaline bread, it is a partially digested farinaceous food, palatable, and highly nutritious. Before being placed in the oven sufficient time is allowed for the partial conversion of the starch into dextrin and maltose by the action of the malt ferment. The bread is thus rendered highly suitable for those of impaired or weak digestion, and can in part be directly absorbed from the stomach before the natural intestinal processes of digestion have had an opportunity of coming into play. The infant rusks are made on the same principle, that is to say, they are partially digested. Soaked in milk they form an excellent food for infants as soon as they can give up an exclusive milk diet, and constitute a useful stepping stone to less easily digested farinaceous food, such as bread and milk, arrowroot, gruel, &c. The sample of bermaline malt we have examined, and find of excellent quality, possessing nutritive and digestive properties of high merit. For persons or children who require fattening, or in whom the natural processes of digestion, are feeble, it constitutes an admirable addition to the ordinary dietary. It may be taken in quantities of a tablespoonful at a time during or immediately after meals, either plain or mixed with milk or water.

## Figures and Tables

**Figure f1:**